# Thermal performance of *Wolffia globosa* under climate change: heatwaves impair population growth

**DOI:** 10.1093/aobpla/plaf068

**Published:** 2026-03-11

**Authors:** Kim Cuddington, Melanie Kuntze, Debora Andrade-Pereira, Yvan Gasmen, Jiayi Wu, Ashley Ferns, Xuewen Geng

**Affiliations:** Department of Biology, University of Waterloo, Waterloo, ON, Canada N2L 3G1; Department of Animal BioSciences, University of Guelph, Guelph, ON, Canada N1G 2W1; Department of Biology, University of Waterloo, Waterloo, ON, Canada N2L 3G1; Department of Biology, University of Waterloo, Waterloo, ON, Canada N2L 3G1; Department of Biology, University of Waterloo, Waterloo, ON, Canada N2L 3G1; Department of Biology, University of Waterloo, Waterloo, ON, Canada N2L 3G1; Department of Biology, University of Waterloo, Waterloo, ON, Canada N2L 3G1

**Keywords:** *Wolffia globosa* (Roxb.) Hartog & Plas, thermal performance, climate change

## Abstract

Climate change impacts on temperature may alter the availability of plants used for food. Some species may have asymmetric responses to temperature, with growth rates that fall rapidly at temperatures above the optimum. As a result, even if mean temperatures increase towards optimal conditions, fluctuations about this mean can substantially decrease growth. We use *Wolffia globosa*, a tropical duckweed harvested for food in Southeast Asia, to examine the impacts of predicted changes in temperatures. This aquatic plant has a fast growth rate, a high protein content, and is also a source of important nutrients. Therefore, it could play an important role in food security under climate change. For constant temperatures there is no significant difference between growth at current conditions and those predicted in the next 40 years according to the high emissions scenario (SSP5-8.5 scenario) in Thailand, Laos and Myanmar. However, when temperatures are allowed to fluctuate about the mean in a pattern similar to recent heatwave conditions in Thailand, we find significantly lower growth rates at the optimum than at current mean temperatures. This decrease is driven by an increase in frond death at higher temperatures. Nevertheless, given the fast growth rate of this species relative to other food crops, and the mitigating impact of water on the magnitude of temperature fluctuations, it seems likely that *W. globosa* may more rapidly recover from extreme heat events than other crop species, and is therefore a suitable candidate for adapting food systems to climate change impacts.

## Teaser

We present the first thermal performance curve for the population growth rates of Wolffia globosa, a high protein aquatic plant harvested for food in SouthEast Asia. Optimal constant temperatures are quite high, and produce very fast population growth. However, we find that the asymmetric thermal response interacts with temperature variation during heatwave conditions. Therefore, changes to mean and variance of temperatures under climate change are likely to cause population crashes.

## Introduction

In many regions, climate change is expected to increase both the mean and variance of air temperatures, simultaneously generating a higher frequency of heatwaves. Predictions regarding the potential impacts of these changes on plants used for food can be made using thermal response studies that measure aspects of performance, such as relative growth rates or yield, as a function of temperature. The probable negative impacts on many species and concurrent human population growth both point to the need to identify species that may help with future food security. We examined the effects of temperature on the growth rates of *Wolffia globosa*, an aquatic plant that is a good source of protein and other nutrients. We determined optimal growing temperatures and examined the probable impacts of changes in temperature on its use in southeast Asia.

Thermal performance curves fit to growth rates have been used to predict potential climate change impacts for various agricultural species (e.g. Agnolucci et al. 2020). In general, these curves are asymmetric with an exponential increase up to some optimal mean temperature, beyond which there is an abrupt decrease (Huey and Berrigan 2001, Amarasekare and Savage 2012, Rezende and Bozinovic 2019). While in some cases climate change might be predicted to increase plant performance by moving mean temperatures closer to a performance optimum, we must also include the impacts of variation. As a result of the curve asymmetry, temperature fluctuations will have different effects depending on their magnitude and the location of the mean relative to the optimum (Ruel and Ayres 1999, Estay et al. 2014, Denny 2019). We expect large negative impacts when there is variation at or above optimal temperatures, but small increases in performance with variation at cooler temperatures. Given the asymmetry of most thermal performance curves, it seems probable that deleterious impacts from heatwave events will outweigh any gains in performance as temperatures increase.

**Figure 1:**
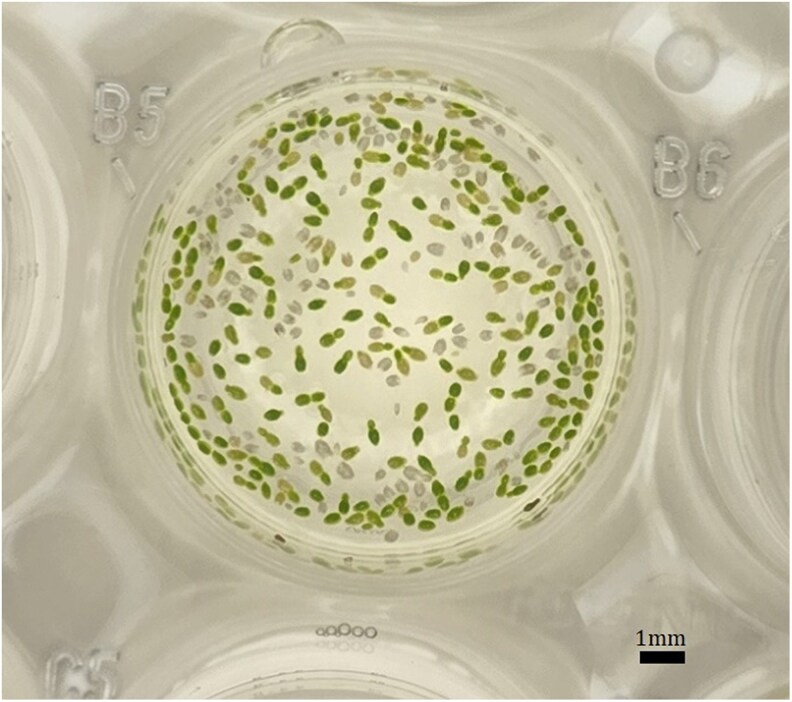
*Wofflia globosa* in a single well of the 24-well plate used during experiments. Green fronds are live and white or faded fronds are dead. Each well could accommodate about 190 plants. Note that the density of fronds pictured here is much larger than that observed during experimental treatments.

We considered the impact of probable changes to temperature mean and variance on the performance of *W. globosa*, a native of Southeast Asia (Landolt 1986). This species is a small, fast-growing aquatic plant that primarily reproduces asexually (Fig. 1). Like other duckweed species, individual plants are recognized by their highly simplified, oval-shaped stem known as a frond that floats on the surface of calm freshwater in ponds, lakes, and marshes (Vu et al. 2020). *W. globosa* has traditionally been used as a food source in these regions, and is primarily harvested, rather than cultivated, twice a week from November to July (European Food Safety Authority (EFSA) 2021). The species has a very fast growth rate and a higher protein content than wheat, corn, brown rice and peas (Boonarsa et al. 2024), which suggests that it could play an important role in sustainable food security (e.g. Nirmal et al. 2025).

The potential impacts of climate change on the growth and yield of *W. globosa* in its native range are unclear. Air temperatures are expected to increase by about 1.5°C–2°C during *W. globosa*’s reproductive months in Thailand, Myanmar (Burma) and Laos in the next 40 years according to the high emissions scenario (SSP5-8.5 scenario; IPCC 2023). We also know that some important food crops may be negatively affected by rising mean temperatures (Knox et al. 2012, Bandara and Cai 2014). Changes to temperature variance and timing are also predicted to increase the frequency of heatwaves (e.g. Meehl and Tebaldi 2004). Heatwaves and associated droughts can significantly impact crop yield by decreasing seed size and number, especially when heat events occur during reproductive stages (Hatfield et al. 2011, Cohen et al. 2021).

Since *W. globosa* is aquatic, predicted impacts on air temperatures must be interpreted in terms of changes to the surface temperatures of the small bodies of water where it is found. In general, small water bodies are closely influenced by air temperature (Jacobs et al. 2008). The correlation between air temperature and water temperature may drive the global average temperature of freshwater system to rise by 0.163°C per decade (Liu et al. 2020). For example, a regression model using air temperature was able to predict water temperature in Pakkok River in Thailand, and suggested that increasing air temperature may cause an increase of 1°C in water temperature with air temperature warming of >4°C (Tongnunui et al. 2023).

While it is known that temperature is an important driver in duckweed growth rates, *Wolffia* spp. are the among the least studied genera in this group (Pasos-Panqueva et al. 2024). We found no thermal performance curves in literature searches across several databases, including Thai databases, and other reports suggest that there is very limited information about how possible temperature increases and fluctuations will impact the growth rates of *W. globosa* (EFSA 2021). There are published estimates of growth rates measured at single temperatures between 25°C and 30°C (e.g. Sree et al. 2015, Romano and Aronne 2021); however, since thermal performance is nonlinear, point estimates of growth rates cannot be used to determine growth rates at higher temperatures. Based on studies on related species such as *Wolffia punctata*, we hypothesize that the optimal temperature for this species may be quite high (∼30°C), and that the thermal performance curve of *W. globosa* may have a similar asymmetrical shape as these species, with a sharp drop from the optimal temperature to the upper critical temperature (Docauer 1983).

To predict how future increases in temperature in Southeast Asia will impact the use of *W. globosa* as a harvested food source, we will measure the relative population growth rates of plants over a range of temperatures, and compare its performance at current and predicted mean temperatures in Thailand. To determine the likely shape of the temperature response, we will assess the fit of several thermal performance curves to the measured population growth rates. If the best fit curve is asymmetric, then we predict the relative growth rates will decrease at high mean temperatures when there is variance (Slein et al. 2023). In particular, a very sharp asymmetry at higher temperatures could lead to catastrophic impacts of heatwaves. To test this prediction, we will also compare population growth rates when temperature is allowed to fluctuate similarly to current normal conditions, and under conditions that mimic a recent heatwave in Thailand.

## Materials and methods

### Strain origin and maintenance

Strains of *W. globosa* (accession number 8692) originally collected in Kyushu, Fukuoka, Chikugo, Japan, and curated and stored at Rutgers Duckweed Stock Cooperative (RDSC) were acquired in May 2022 and maintained aseptically. While we were unable to obtain clones from Thailand, we note that population-level thermal performance curves generally reflect species-level niche breadth reasonably well (Wooliver et al. 2022). In addition, there is generally similarity between thermal performance curves of different genotypes in duckweeds (Usui and Angert 2024, Supplementary Figure S5), and most estimates of the relative growth rate of *W. globosa* at 25°C are quite similar across clones (see Sree et al. 2015).

Plants were maintained in 20°C Memmert incubators (IPP-500) and cultivated on a modified Hoagland's medium, a macronutrient solution commonly used for culturing duckweed species (e.g. Fang et al. 2020). A 16:8 light-dark photoperiod was applied using LED lights (Kema Keur 0J-304 250V-3A 3A/125VAC E354212) to provide PAR intensity between 78.0 and 91.7 µmol m^−2^ s^−1^, which is a moderate level for the growth of duckweeds under controlled conditions (Pasos-Panqueva et al. 2024). About every 20 days, we created a new source population using new growth media to ensure that plants selected for experiments maintained active metabolic rates.

### Temperature treatments

We used a range of constant temperatures (14.0°C, 20.0°C, 25.0°C, 26.5°C, 28.5°C, 31.5°C, 37°C) to construct a thermal performance curve. The lowest temperature tested, 14°C, was selected because it corresponds to the threshold at which similar species typically enter dormancy, producing inactive structures called turions (Romano and Aronne 2021). We selected intermediate temperatures to allow comparison to both previously published work (e.g. Ziegler et al. 2015), and to match current and predicted mean temperatures during the growth season in Thailand, as well as mean temperatures during a recent heatwave (see below for description). The highest temperature of 37°C was chosen based on the upper critical threshold for two other tropical species, *W. punctata* and *Wolffia columbiana* (Docauer 1983), and is similar to the median maximum temperature observed during a recent heatwave (36.7°C, see below).

To assess how predicted temperature increases will impact *W. globosa* harvests, we focused on Thailand's current and future climate, as this country has a longstanding tradition of *W. globosa* consumption (Siriwat et al. 2023). We used monthly historical (1991–2020) and predicted (2040–2059) average temperatures from the World Bank Climate Change Knowledge Portal (2024), as data at finer temporal scales is not available. Within this portal, predicted temperatures are based on the 2023 Coupled Model Intercomparison Projects (CMIPs) from the Intergovernmental Panel on Climate Change (IPCC 2023).

We identified the current average temperature during the growing season (November to July), the period when *W. globosa* is predominantly used as a food source (EFSA Panel on Nutrition Novel Foods and Food Allergens et al. 2021). This value represents the mean of the monthly 50th percentile (median) temperatures for that period. Next, we applied the SSP5-8.5 climate change scenario, which is a business-as-usual emissions prediction without additional mitigation efforts (IPCC 2023), to estimate the average temperature increase in Southeast Asia. By averaging the predicted monthly 50th percentile (median) temperatures temperature increases for the growing season months, we determined that mean temperatures are expected to rise by approximately 2°C over the next 20–40 years, raising Thailand's average growing season temperature from 26.5°C to 28.5°C (The World Bank Group 2024).

To assess how warmer temperatures with natural variation might impact *W. globosa* performance, we also created two fluctuating treatments based on the following reference scenarios: (1) mean current temperature (26.5°C) and (2) mean temperature (31.5°C) during a heatwave in Thailand from April 22 to 23 May 2024 (Visual Crossing Corporation 2024, see Supplementary Information S1). For the mean current temperature, we obtained the average minimum and maximum monthly temperatures for the November–July period. For the heat wave scenario, we used the average hourly minimum and maximum temperatures, along with the overall hourly average temperature. We adjusted the minimum and maximum temperatures to ensure that the average temperatures in the fluctuating treatments matched those of the constant temperature treatments, while preserving a consistent standard deviation of 3.2°C–3.3°C across the two fluctuating treatments (Table 1).

**Table 1:** Temperature parameters, based on historical and predicted temperatures in Thailand, used to create fluctuating thermal sequences for each treatment: season average and heatwave.

Temperature	Season average	Heatwave
Minimum	21.2°C (22°C)	28.3°C (27.1°C)
Mean	26.5°C	31.5°C
Maximum	31.9°C (31°C)	36.1°C (36°C)
Standard deviation^a^	3.3°C	3.2°C

^a^Standard deviation values were calculated based on thermal sequences generated using the adjusted minimum and maximum temperatures (listed in parentheses).

**Figure 2:**
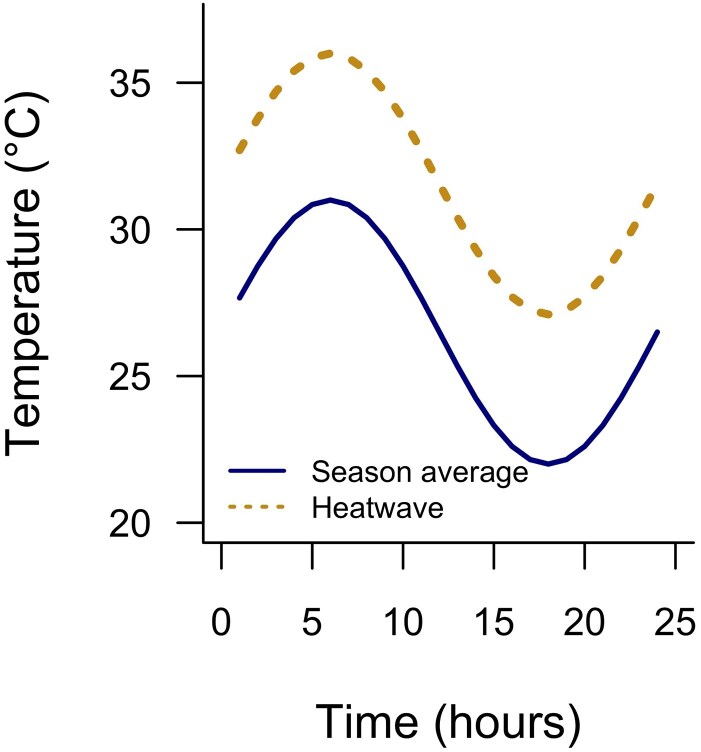
Daily fluctuation for variable temperature treatments. The season average treatment reflects the current growing season temperatures in Thailand, and heatwave treatment mean and variance are based on an actual heat wave event that occurred from April–May 2024.

We used the adjusted maximum and minimum temperatures to create fluctuating temperature treatments using a sinusoidal function, a commonly employed method for simulating temperature cycles (National Institute of Water and Atmospheric Research (NIWA) 2023), using the following equation: *T*(hour) = *T*_min_ + (*T*_max_ − *T*_min_) × (0.5 *+* 0.5sin(2*π*(hour)/24)), where *T*(hour) is the temperature at a given hour, hour is the index representing each hourly time step, and *T*_min_ and *T*_max_ are the minimum and maximum temperatures in each scenario. The sinusoidal function generates thermal sequences with hourly temperatures by interpolating between the minimum and maximum using a sine wave, ensuring smooth transitions over a 24-h period to mimic natural daily temperature fluctuations. For each treatment, we repeated this pattern daily for a total of 5 days (Fig. 2).

### Experimental methods for temperature treatments

The experiments were conducted over a 5-day (120-h) period, which was sufficient to observe a change in the number of fronds. *W. globosa* has an extremely fast growth rate with a doubling time of 1–2 days (Appenroth et al. 2018). In addition, this experimental duration is similar to that used in previous studies of the effects of temperature on growth rates of *Wolffia* spp. (7 days in Sree et al. 2015). Temperature treatments were conducted in the previously described incubators, using the proprietary software Celsius (version 10.1.4) to implement the desired sequence of temperatures. Each replicate consisted of twelve pairs of fronds randomly selected from a mixed-age source population of *W. globosa* and placed into a 24-well plate. Each well contained about 1000 µl of Hoagland’s solution, and could accommodate ∼190 fronds, given an estimated frond surface area of ∼0.5–1 mm². Therefore, considering the short duration of our experiments, well size posed no limitations to growth. Replicates of each temperature treatment were randomly assigned to individual incubators. For each temperature treatment, a minimum of five replicates were completed.

At the start and conclusion of the 5-day experiment, a Zeiss Stemi 2000-C stereomicroscope was used to examine frond health and perform count fronds for each replicate. This visual inspection was used to classify fronds as alive if they exhibited less than 90% loss of green pigmentation (Thomson and Dennis 2013); otherwise, they were considered dead. Fronds of all sizes were included, and both live and dead fronds were counted. The relative growth rate was then calculated as (ln(live fronds) − ln(original number of fronds))/duration.

### Data analysis

We fit three thermal performance curves to the relative growth rate across all constant temperature treatments. We used a modified Ratkowsky model as: (*a*(*T* − *T*_min_))^2^(1 − *e*(*b*(*T* − *T*_max_))), where *T* is the temperature, *T*_min_ is the low temperature where rate of performance becomes 0, *T*_max_ is the high temperature where rate of performance becomes negative, and *a* and *b* are rate parameters (Ratkowsky et al. 1983). We also fit a Brière2 curve (Brière et al. 1999) given as: *aT*(*T* − *T*_min_)(*T*_max_ − *T*)^(1/*b*), and a simple quadratic curve. We fit these curves using the R package *nls-multstart* (Padfield and Matheson 2023) where minimum and maximum values for initial coefficients were *T*_min_ = (−15, 5), *T*_max_ = (14, 37), *a* = (−0.5, 1.5), and *b* = (−1, 1.1), for both the Ratkowsky and Biere2 functions, and *a* = (−2, 2), *b* = (−2, 2), and *c* = (−1, 1) for the quadratic.

We used generalized linear models with a Poisson distribution to separately analyze the impact of temperature treatments on the number of live and dead fronds, where replicates were randomly subsampled to ensure an equal number in each temperature treatment. We used this test to determine if there were significant differences in the number of live or dead fronds at different constant temperatures. In addition, we tested for significant differences in the number of live fronds in constant or fluctuating treatments at 26.5°C and 31.5°C. All analyses were performed using R Statistical Software (v3.6.3; R Core Team 2020), using the *eemeans* package (Russell 2021) for pairwise comparisons of each treatment with a Tukey’s adjustment for multiple tests.

## Results

**Figure 3:**
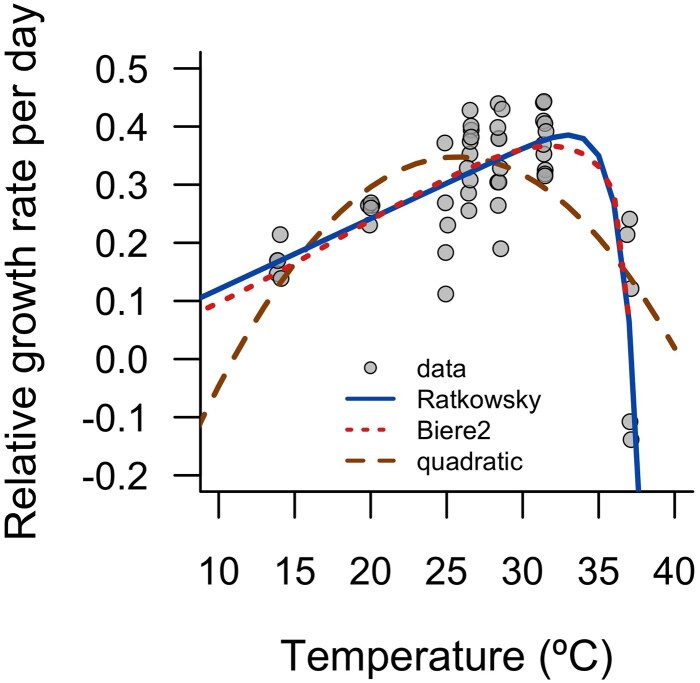
Modified Ratkowsky, Brière2, and quadratic functions fit to the relative growth rate of *W. globosa* measured at different constant temperatures. The modified Ratkowsky curve has the best fit with parameters *a* = 0.014, *b* = 0.43, *T*_min_ = −15°C, and *T*_max_ = 37.3°C. A small amount of jitter has been added to the data to improve clarity.

The best fit thermal performance curve at constant temperatures possesses a clear asymmetrical shape, with a drastic decline in growth rate towards the higher temperatures, and a suggested optimal temperature of ∼32°C (Fig. 3). The best fit curve for the relative growth rate was the modified Ratkowsky function as indicated by AIC (Ratkowsky: −102.4, Brière2: −101.8 and quadratic: −85.3) and the residual sum of squares (Ratkowsky: 0.31, Brière2: 0.31 and quadratic: 0.45). Although the difference in the fit of the Ratkowsky and Brière2 curves is negligible.

**Figure 4:**
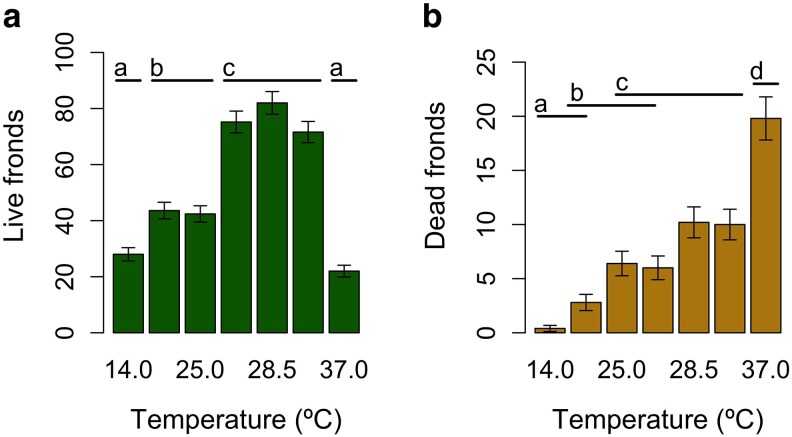
Mean and standard error calculated using a Poisson distribution of the (a) number of live fronds or (b) number of dead fronds of *W. globosa*, where there are five replicates at each of the different constant temperatures. Horizontal bars and letters indicate temperature treatments that are not significantly different.

Temperature affected the number of live fronds after 5 days (Fig. 4a). The number of fronds in the lowest (14°C) and highest (37°C) constant temperature treatments was significantly smaller than all other temperature treatments (all pairwise comparisons had *P* < 0.001), but was not significantly different between these two treatments (*n* = 10, *z* = 1.89, *P* = 0.49). However, there was no significant difference between the number of fronds in treatments with temperatures at the current mean during the growing season in Thailand (26.5°C) and the projected mean in 40 years (28.5°C) (*n* = 10, *z* = −1.07, *P* = 0.94). We note that, even at optimal temperatures, the number of fronds in a single well was less than half of the potential capacity.

There was a significant impact of temperature on the number of dead fronds. Although the number of live fronds did not differ in the coldest and hottest temperature treatments (see above), analysis of the number of dead fronds clearly indicated that there were different mechanisms at work: reduced reproductive rate at cold temperatures and increased death rate at high temperatures. The largest number of dead fronds was found in the hottest treatment (37°C, all pairwise comparisons *P* < 0.05) and the smallest number was in the two coldest treatments (14°C and 20°C; these two cold treatments were not significantly different; *n* = 10, *z* = −2.57, *P* = 0.13) (Fig. 4b). Again, there was no difference in the number of dead fronds at the current mean temperatures during the growing season in Thailand (26.5°C) and the projected mean in 20–40 years (28.5°C) (*n* = 10, *z* = −1.39, *P* = 0.81).

**Figure 5:**
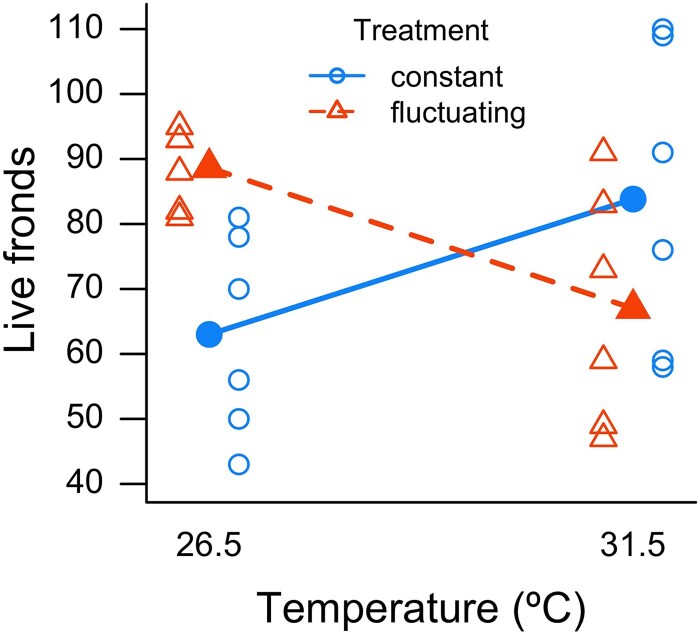
Interaction plot of the live fronds of *W. globosa* where there are six replicates for each treatment. Temperatures are either constant (solid blue lines and circles), or fluctuating (dashed red lines and triangles). Treatment means are indicated by filled points.

We note, however, that constant temperature treatments may not reflect the impact of naturally fluctuating temperature regimes. Comparisons between the number of live fronds in either constant or fluctuating temperature treatments at either 26.5°C or 31.5°C indicated that there was a significant interaction (*n* = 24, *z* = −4.40, *P* < 0.0001) between temperature and constant vs fluctuating regimes (Fig. 5). The number of live fronds was lower in the 31.5°C temperature treatment when fluctuations were present, but higher under constant conditions.

## Discussion

We find a difference in the population growth rates of *W. globosa* under constant or fluctuating conditions at mean temperatures representative of predicted warming in Southeast Asia. The relative growth rates of *W. globosa* have an optimum at higher temperatures than expected and so it might be predicted increases in mean temperatures could have a beneficial impact. However, the best fit thermal performance curve is quite nonlinear and asymmetric, with substantial decreases in growth at temperatures higher than the optimum. At constant temperatures reflective of maximums during recent heatwaves, relative growth rate was reduced because of higher frond death rates. At cooler fluctuating temperatures, the asymmetric shape of the thermal performance curve caused greater reductions in growth near the optimum temperature. Under natural variation higher growth rates are expected at temperatures cooler than the optimum under constant conditions. Therefore, it is likely that future climate change impacts, such as increased frequency of heatwaves and increased mean water temperatures, may have a negative impact on the harvesting of the species for food.

The highest measured relative growth rates at constant temperatures were at 31.5°C. Growth rates range from a minimum of 0.17 ± 0.033 per day (at 14°C) to a maximum of 0.38 ± 0.023 (at 31.5°C) per day, and are similar to previously published rates for *W. globosa*. Sree et al. (2015) report relative growth rates ranging from 0.155 ± 0.040 per day to 0.559 ± 0.003, at 25°C ± 1°C for eight clones (some data originally from Ziegler et al. 2015). While there was some large variation, particularly between clones from South America vs Asia, most clones had growth rates close to the mean of 0.374 fronds per day. Similarly, Romano et al. (2024) give a value of ∼0.38 per day at 30°C ± 0.5°C. However, we note that growth rates are highly dependent on the particular clone, nutrients and light levels (e.g. Sree et al. 2015), which vary amongst these studies. In addition, all these studies, and our own, were designed to examine current responses of plants acclimated to moderate temperatures rather than any potential adaptations following longer exposure to colder or hotter conditions.

We provide the first thermal performance curve for *W. globosa* over a range of constant temperatures from 14.0°C to 37.5°C. Thermal performance curves can be valuable for gaining insight into how organisms respond to climate warming and variability. Because most physiological functions determining performance follow a similar curved and asymmetric shape, such curves allow us to predict when rising mean temperatures and fluctuations around them are likely to be beneficial, neutral, or detrimental, depending on proximity to thermal optima (Ruel and Ayres 1999). For *W. globosa*, the best fit curves have a typical asymmetric shape, rather than symmetric one, with slow decline in relative growth rates as the temperature becomes colder, but an abrupt decrease in performance at warmer temperatures (Fig. 3). Both fitted asymmetric curves suggest that, at constant temperatures, the maximum relative growth rate occurs at ∼32°C, while growth decreases below 26.52°C and above 32°C. More measurements at temperatures greater than 31.5°C would increase our confidence in this estimated optimum.

The thermal performance for *W. globosa* is quite similar to that of some other species of duckweed, such as *W. punctata* and *W. columbiana* (Docauer 1983). These species also have an asymmetric response to temperature with an optimum near 30°C and an abrupt decrease with an upper critical threshold near 37°C (Docauer 1983). These responses differ from more symmetric shapes, such as those reported for other duckweeds like *Lemna minor* (Andrade-Pereira and Cuddington 2024) and similar floating plants like *Azolla* spp. (Ocloo et al. 2023).

An analysis of the number of live and dead fronds reveals that there are different causes for the decreased relative growth rates at low and high temperatures. While there is no difference in the number of live fronds at the highest and lowest temperatures tested, there were significantly more dead fronds at the highest temperature. In fact, at the coldest temperature there were nearly zero dead fronds. Therefore, the reduced relative growth rates at cold temperatures are primarily due to reduced reproduction, while the lower or negative growth rates at hot temperatures are caused by an increased death rate. While higher temperatures are known to reduce duckweed lifespan (Paiha 2021), the short length of our experiments (5 days compared to the average lifespan of 14–20 days Ruekaewma 2011, reported in EFSA 2021) and the large numbers of dead fronds both point to temperature-dependent mortality rather than accelerated senescence as the probable cause of the reduced growth rates at the highest temperature.

Our thermal performance curve with constant temperatures could suggest that there will be no significant near-term impacts of climate change on the growth of *W. globosa* in the regions where it is currently harvested. We find no significant difference in the growth of *W. globosa* at the current mean air temperature during the growing season in Thailand (26.5°C) and the projected mean in 40 years (28.5°C) of the 2023 Coupled Model Intercomparison Projects (CMIPs) from the Intergovernmental Panel on Climate Change (IPCC 2023). In comparison, the predicted increase in temperature in Laos and Myanmar, from 23.5°C to 25°C (The World Bank Group 2024), will bring mean temperatures closer to *W. globosa*’s optimum; therefore possibly increasing its growth rate. Over a longer timeframe, under the high-emission SSP5-8.5 scenario for Southeast Asia, projections suggest that average July surface air temperatures in the region could rise to 27.62°C by 2050 and 29.13°C by 2100 (Sentian et al. 2022), but this is still within the range of optimal performance.

However, when there is a nonlinear thermal response with an abrupt decrease, even small variance in temperature conditions can decrease performance (Slein et al. 2023). We found a significant interaction when we compared constant and fluctuating temperature regimes. When temperatures were allowed to fluctuate, the relative growth rate was higher at 26.5°C rather than 31.5°C. Therefore, it is possible that when a realistic level of temperature fluctuation is considered, climate change may result in growth decreases. This conclusion is reinforced when we consider that a greater frequency of heatwaves is anticipated under future conditions (Meehl and Tebaldi 2004). Thailand’s temperature fluctuations and extremes on a local scale are almost linearly related to large-scale or global mean temperature changes (Amnuaylojaroen and Parasin 2022). The mean and degree of variance of the 31.5°C fluctuating treatment was based on actual air temperatures experienced during the 2024 heatwave in Thailand. This temperature treatment had mean conditions not very different from both the mean water temperatures in fish ponds in May (Sriyasak et al. 2013), and the projected summer mean air temperature for Thailand in 2080 (30.63°C, SSP5-8.5; The World Bank 2024). Therefore, if current emission trends persist, temperature conditions similar to our heatwave treatment will increase, and growth rates may decline.

At the water surface where *W. globosa* grows, we might expect mean temperatures to be very closely linked to mean air temperatures (Jacobs et al. 2008). However, the variance of air temperatures is usually larger than water temperatures because the high specific heat capacity of water dampens fluctuations. In addition, in deeper ponds where stratification is possible, water temperatures may be cooler than air temperatures. For example, a study of 18 fish ponds with depths of 0.8–2.0 m in northern Thailand reports that during the dry season in January, mean air temperature was warmer (28.3°C ± 4.11°C) and had a much wider range (6.5°C−35.83°C) than water temperatures (mean of 26.3°C ± 0.57°C with range from 25.5°C to 27.1°C) (Sriyasak et al. 2013). The rainy season decreases stratification, and in May, water temperatures were warmer than air temperatures (mean air temperature: 28.14°C ± 4.03°C; mean water temperature: 30.44°C ± 0.80°C). Therefore, water temperatures always had lower variance, and the constant temperatures used to construct the thermal performance curve may reasonably reflect this lack of fluctuation.

The mean water temperatures reported during the rainy season are right at the optimum for *W. globosa*, and so our interpretation differs once we consider natural fluctuations. At these temperatures, even a small amount of variance could lead to abrupt decreases in growth rates. Moreover, in the Lower Songkhram River regions, where *W. globosa* is used for food (Norkum Ai et al. 2025), water temperatures may rise beyond the optimum (e.g. 35°C) during the growing season (Satrawaha et al. 2009). Future research on the thermal performance of *W. globosa* should include information on seasonal differences in both the mean and variance of water temperatures at the surface of these small water bodies.

This information regarding the temperature dependence of the relative growth rate of *W. globosa* has implications for both indoor cultivation and also its spread in non-native regions. Recently there has been increased interest in commercial production of this species, and for its use in space travel (Romano and Aronne 2021). For example, some food suppliers opened a novel food dossier for both *W. globosa* and *W. arrhizi*, and the consumption of *W. globosa* was approved by the European Food Safety Authority. Recommendations regarding cultivation typically suggest a broad range of conditions from 20°C to 30°C as optimal (Kaur 2023), but our studies indicate that maximum growth will occur in a narrower and warmer range from 26.5°C to 32°C. Nevertheless, it is clear that the species has positive growth rates at the coolest temperature measured (14°C), and therefore, it is likely to continue its spread through regions of Europe and North America with moderate temperatures (e.g. UK: Lansdown et al. 2022, Germany: Frank et al. 2020, France: Lecron et al. 2021, Czech Republic: Vávra et al. 2024), where like other invasive duckweeds, it may have negative impacts (e.g. Ceschin et al. 2020).

Other impacts of climate change, not considered here, may also decrease population growth. Drought is associated with climate change and heatwaves (Khadka et al. 2024) and may impact the occurrence of ponds and small waterbodies that are required for growth. Intraspecies competition with other surface-covering plant species, such as algae, cyanobacteria, and other duckweeds is common (Coughlan et al. 2022, Ziegler et al. 2023), and may increase with climate change. For example, Gillies et al. (2024) find that high-temperature stress could increase sensitivity to competition in *Lemna* duckweed species.

Predictions for other crop species in this region, such as rice and corn, similarly suggest that near term increases in mean temperatures may benefit growth rates, but that extreme conditions would decrease yield (e.g. Amnuaylojaroen and Parasin 2022, Waqas et al. 2025). While *W. globosa* is likely to be negatively impacted by larger increases in mean temperatures and associated heatwaves, we should note that its very high growth rate at average high temperatures will enable quick recovery from extreme conditions. Depending on the timing of the event, crops with comparable protein, such as peas or soy may have a reduction or complete loss of harvest for the season (e.g. Siebers et al. 2015). In contrast, as long as a small proportion of the fronds survive, *W. globosa* has the potential for ample future harvests in a few weeks.

## Conclusion

There are a limited number of studies evaluating thermal performance curves of plants (Wooliver et al. 2022), even though such data may provide crucial information for climate change adaptation. We provide the first thermal performance curve of the relative growth rates of *W. globosa*, and use this information to predict responses to climate change in Southeast Asia where it is currently harvested. While performance under constant temperature conditions suggests little impact under climate change, we find decreased growth rates when temperatures fluctuate, and note that the highly asymmetric thermal performance curve can lead to sudden catastrophic impacts at high temperatures. Nevertheless, the fast growth rate of the species suggests fast recovery from heat events is possible. This potential for resilience and the excellent nutritional profile of the species suggest it could play an important role in climate change adaptation.

## Supplementary Material

plaf068_Supplementary_Data

## Data Availability

Raw data and R code are available online at https://github.com/Cuddington-Lab/Wolffia_AoBPlants/tree/main.

## References

[plaf068-B1] Agnolucci P, Rapti C, Alexander P et al Impacts of rising temperatures and farm management practices on global yields of 18 crops. Nat Food 2020;1:562–71. 10.1038/s43016-020-00148-x 37128016

[plaf068-B2] Amarasekare P, Savage V. A framework for elucidating the temperature dependence of fitness. Am Nat 2012;179:178–91. 10.1086/663677 22218308

[plaf068-B3] Amnuaylojaroen T, Parasin N. The future extreme temperature under RCP8.5 reduces the yields of major crops in northern peninsular of Southeast Asia. Scientific World Journal 2022;2022:1410849. 10.1155/2022/1410849 35401057 PMC8986419

[plaf068-B4] Andrade-Pereira D, Cuddington K. Range expansion risk for a newly established invasive duckweed species in Europe and Canada. Plant Ecol 2024;225:839–50. 10.1007/s11258-024-01436-3

[plaf068-B5] Appenroth K-J, Sree KS, Bog M et al Nutritional value of the duckweed species of the genus Wolffia (Lemnaceae) as human food. Front Chem 2018;6:483. 10.3389/fchem.2018.00483/full 30420949 PMC6215809

[plaf068-B6] Bandara JS, Cai Y. The impact of climate change on food crop productivity, food prices and food security in South Asia. Econ Anal Policy 2014;44:451–65. 10.1016/j.eap.2014.09.005

[plaf068-B7] Boonarsa P, Bunyatratchata A, Chumroenphat T et al Nutritional quality, functional properties, and biological characterization of watermeal (*Wolffia globosa*). Horticulturae 2024;10:1171. 10.3390/horticulturae10111171

[plaf068-B8] Brière JF, Pracros P, Roux L et al A novel rate model of temperature-dependent development for arthropods. Environ Entomol 1999;28:22–9. 10.1093/ee/28.1.22

[plaf068-B9] Ceschin S, Ferrante G, Mariani F et al Habitat change and alteration of plant and invertebrate communities in waterbodies dominated by the invasive alien macrophyte *Lemna minuta* Kunth. Biol Invasions 2020;22:1325–37. 10.1007/s10530-019-02185-5

[plaf068-B10] Cohen I, Zandalinas SI, Huck C et al Meta-analysis of drought and heat stress combination impact on crop yield and yield components. Physiol Plant 2021;171:66–76. 10.1111/ppl.13203 32880977

[plaf068-B11] Coughlan NE, Walsh É, Bolger P et al Duckweed bioreactors: challenges and opportunities for large-scale indoor cultivation of lemnaceae. J Clean Prod 2022;336:130285. 10.1016/j.jclepro.2021.130285

[plaf068-B12] Denny M . Performance in a variable world: using Jensen’s inequality to scale up from individuals to populations. Conserv Physiol 2019;7:coz053. 10.1093/conphys/coz053 31528348 PMC6736373

[plaf068-B13] Docauer DM . A nutrient basis for the distribution of the Lemnaceae. Ph.D. Thesis, University of Michigan, USA. 1983. https://www.proquest.com/docview/303177608/abstract/29AAE44BAFE44326PQ/1

[plaf068-B14] Estay SA, Lima M, Bozinovic F. The role of temperature variability on insect performance and population dynamics in a warming world. Oikos 2014;123:131–40. 10.1111/j.1600-0706.2013.00607.x

[plaf068-B15] European Food Safety Authority (EFSA) . Technical report on the notification of fresh plants of *Wolffia arrhiza* and *Wolffia globosa* as a traditional food from a third country pursuant to Article 14 of Regulation (EU) 2015/2283. EFSA Support Publ 2021;18:6658E. 10.2903/sp.efsa.2021.EN-6658

[plaf068-B16] Fang Y, Du A, Tan L et al The transcriptome in *Landoltia punctata*. In: Cao X, Fourounjian P, Wang W (eds.) The Duckweed Genomes. Compendium of Plant Genomes. Cham: Springer, 2020, 125–31. 10.1007/978-3-030-11045-1_12

[plaf068-B17] Frank D, Bog M, Appenroth K-J et al Man sieht nur was man kennt—Drei Zwergwasserlinsen-Arten der Gattung Wolffia Schleid. In Sachsen-Anhalt nachgewiesen [The unknown stays mostly unseen. Three species of rootless duckweed (genus Wolffia Schleid.) Found in Saxony-Anhalt; in German]. Mitteilungen zur floristischen Kartierung in Sachsen-Anhalt 2020;25:3–17. 10.21248/mfk.33

[plaf068-B18] Gillies GJ, Angert AL, Usui T. Temperature dependence and genetic variation in resource acquisition strategies in a model freshwater plant. Funct Ecol 2024;38:1600–10. 10.1111/1365-2435.14567

[plaf068-B19] Hatfield JL, Boote KJ, Kimball BA et al Climate impacts on agriculture: implications for crop production. Agron J 2011;103:351–70. 10.2134/agronj2010.0303

[plaf068-B20] Huey RB, Berrigan D. Temperature, demography, and ectotherm fitness. Am Nat 2001;158:204–10. 10.1086/321314 18707349

[plaf068-B21] IPCC . Climate change 2023: Synthesis report. In: Lee H, Romero J (eds.), Contribution of Working Groups I, II and III to the Sixth Assessment Report of the Intergovernmental Panel on Climate Change. Geneva, Switzerland: IPCC, 2023, 35–115. 10.59327/IPCC/AR6-9789291691647

[plaf068-B22] Jacobs AFG, Heusinkveld BG, Kraai A et al Diurnal temperature fluctuations in an artificial small shallow water body. Int J Biometeorol 2008;52:271–80. 10.1007/s00484-007-0121-8 17926069 PMC2668566

[plaf068-B23] Kaur S . *Wolffia globosa* ultimate cultivation guide. 2023. https://www.researchgate.net/profile/Simrat-Kaur-8/publication/382397647_Wolffia_Globose_Ultimate_Cultivation_Guide_Wolffia_globosa_Ultimate_Cultivation_Guide/links/669b555a4a172d2988b1e786/Wolffia-Globose-Ultimate-Cultivation-Guide-Wolffia-globosa-Ultimate-Cultivation-Guide.pdf (3 March 2025, date last accessed).

[plaf068-B24] Khadka D, Babel MS, Tingsanchali T et al Evaluating the impacts of climate change and land-use change on future droughts in northeast Thailand. Sci Rep 2024;14:9746. 10.1038/s41598-024-59113-4 38679611 PMC11056375

[plaf068-B25] Knox J, Hess T, Daccache A et al Climate change impacts on crop productivity in Africa and South Asia. Environ Res Lett 2012;7:034032. 10.1088/1748-9326/7/3/034032

[plaf068-B26] Landolt E . Biosystematic investigations in the family of duckweeds (Lemnaceae), (Vol. 2). The family of Lemnaceae—a monographic study. Volume 1. Veröffentlichungen des Geobotanischen Institutes der Eidg. Techn. Hochschule 1986;71:1–563.

[plaf068-B27] Lansdown R, Kitchener G, Jones E. *Wolffia columbiana* and *W. globosa* (Araceae) new to Britain. Br Irish Bot 2022;4:14–26. 10.33928/bib.2022.04.014

[plaf068-B28] Lecron JM, Fisson P, Fried G et al Deux nouvelles espèces de wolffies en France métropolitaine: Wolffia columbiana H. Karst et W. globosa (Roxb.) Hartog & Plas (Araceae). Bull Soc Bot Centre-Ouest 2021;52:129–36. https://www.sbco.fr/pdf/ArtBull/Bull52/SBCO-Bull52-p129-136-Lecron_al-Deux_nouvelles_Wolffies_France.pdf

[plaf068-B29] Liu S, Zhenghui X, Liu B et al Global river water warming due to climate change and anthropogenic heat emission. Glob Planet Change 2020;193:103289. 10.1016/j.gloplacha.2020.103289

[plaf068-B30] Meehl GA, Tebaldi C. More intense, more frequent, and longer lasting heat waves in the 21st century. Science 2004;305:994–7. 10.1126/science.1098704 15310900

[plaf068-B31] National Institute of Water and Atmospheric Research (NIWA) . *Using trigonometric functions to model climate*. 2023. https://niwa.co.nz/using-trigonometric-functions-model-climate (3 March 2025, date last accessed).

[plaf068-B32] Nirmal N, Anyimadu CF, Khanashyam AC et al Alternative protein sources: addressing global food security and environmental sustainability. Sustain Dev 2025;33:3958–69. 10.1002/sd.3338

[plaf068-B33] Norkum Ai P, Inta A, Sommano SR et al Exploring traditional knowledge and potential uses of local freshwater algae and aquatic plants in Thai Wetland communities. Diversity 2025;17:63. https://www.mdpi.com/1424-2818/17/1/63

[plaf068-B34] Ocloo XS, Vazquez-Prokopec GM, Civitello DJ. Mapping current and future habitat suitability of Azolla spp., a biofertilizer for small-scale rice farming in Africa. PLoS One 2023;18:e0291009. 10.1371/journal.pone.0291009 38109403 PMC10727437

[plaf068-B35] Padfield D, Matheson G. nls.multstart: Robust non-linear regression using AIC scores. R package version 1.3.0, 2023. https://CRAN.R-project.org/package=nls.multstart

[plaf068-B36] Paiha AP . Senescence in Duckweed: an interspecific comparison and the influence of temperature. M.Sc. Thesis, University of Lethbridge, Canada. 2021. https://www.proquest.com/docview/2565083299/abstract/7DA55D1BC3D74D19PQ/1

[plaf068-B37] Pasos-Panqueva J, Baker A, Camargo-Valero MA. Unravelling the impact of light, temperature and nutrient dynamics on duckweed growth: a meta-analysis study. J Environ Manage 2024;366:121721. 10.1016/j.jenvman.2024.121721 39018836

[plaf068-B38] Ratkowsky DA, Lowry RK, McMeekin TA et al Model for bacterial culture growth rate throughout the entire biokinetic temperature range. J Bacteriol 1983;154:1222–6. 10.1128/jb.154.3.1222-1226.1983 6853443 PMC217594

[plaf068-B39] R Core Team . R: A Language and Environment for Statistical Computing. Vienna, Austria: R Foundation for Statistical Computing, 2020. https://www.R-project.org/

[plaf068-B40] Rezende EL, Bozinovic F. Thermal performance across levels of biological organization. Philos Trans R Soc B Biol Sci 2019;374:20180549. 10.1098/rstb.2018.0549 PMC6606466 31203764

[plaf068-B41] Romano LE, Aronne G. The world smallest plants (*Wolffia* sp.) as potential species for bioregenerative life support systems in space. Plants 2021;10:1896. 10.3390/plants10091896 34579428 PMC8470744

[plaf068-B42] Romano LE, Van Loon Jjwa, Izzo LG et al Effects of altered gravity on growth and morphology in Wolffia globosa implications for bioregenerative life support systems and space-based agriculture. Sci. Rep 2024;14:410. https://www.nature.com/articles/s41598-023-49680-3 38172193 10.1038/s41598-023-49680-3 PMC10764921

[plaf068-B43] Ruekaewma N . Optimal conditions for production of Khai-nam, *Wolffia globosa*. Ph.D. Dissertation, Faculty of Science, Chulalongkorn University, 2011.

[plaf068-B44] Ruel JJ, Ayres MP. Jensen’s inequality predicts effects of environmental variation. Trends Ecol Evol 1999;14:361–6. 10.1016/S0169-5347(99)01664-X 10441312

[plaf068-B45] Russell VL . emmeans: estimated marginal means, aka least-squares means. R package version 1.6.0, 2021. https://CRAN.R-project.org/package=emmeans

[plaf068-B46] Satrawaha R, Prathepha P, Andrews R et al Fundamental hydrochemical parameters of the Songkhram River in Northeast Thailand: foundation data for the study of an endangered tropical wetland ecosystem. Limnology 2009;10:7–15. 10.1007/s10201-008-0254-4

[plaf068-B47] Sentian J, Payus CM, Herman F et al Climate change scenarios over Southeast Asia. APN Sci Bull 2022;12: 102–122. 10.30852/sb.2022.1927

[plaf068-B48] Siebers MH, Yendrek CR, Drag D et al Heat waves imposed during early pod development in soybean (*Lycine max*) cause significant yield loss despite a rapid recovery from oxidative stress. Glob Chang Biol 2015;21:3114–25. 10.1111/gcb.12935 25845935

[plaf068-B49] Siriwat W, Ungwiwatkul S, Unban K et al Extraction, enzymatic modification, and anti-cancer potential of an alternative plant-based protein from *Wolffia globosa*. Foods 2023;12:3815. 10.3390/foods12203815 37893708 PMC10606862

[plaf068-B50] Slein MA, Bernhardt JR, O’Connor MI et al Effects of thermal fluctuations on biological processes: a meta-analysis of experiments manipulating thermal variability. Proc R Soc Lond B Biol Sci 2023;290:20222225. 10.1098/rspb.2022.2225 PMC9904952 36750193

[plaf068-B51] Sree KS, Sudakaran S, Appenroth K-J. How fast can angiosperms grow? Species and clonal diversity of growth rates in the genus Wolffia (Lemnaceae). Acta Physiol Plant 2015;37:204. 10.1007/s11738-015-1951-3

[plaf068-B52] Sriyasak P, Chitmanat C, Whangchai N et al Effects of temperature upon water turnover in fish ponds in northern Thailand. Int J Geosci 2013;04:18–23. 10.4236/ijg.2013.45B004

[plaf068-B53] The World Bank Group . *Climate change knowledge portal*, 2024. https://climateknowledgeportal.worldbank.org/ (25 February 2025, date last accessed).

[plaf068-B54] Thomson ELS, Dennis JJ. Common duckweed (*Lemna minor*) is a versatile high-throughput infection model for the *Burkholderia cepacia* complex and other pathogenic bacteria. PLoS One 2013;8:e80102. 10.1371/journal.pone.0080102 24223216 PMC3819297

[plaf068-B55] Tongnunui S, Sooksawat T, Chotwiwatthanakun C et al Seasonal changes in upper thermaltolerances of freshwater Thai fishes. Water (Basel) 2023;15:350. 10.3390/w15020350

[plaf068-B56] EFSA Panel on Nutrition, Novel Foods, and Food Allergens (NDA), Turck D, Bohn T et al Safety of *Wolffia globosa* powder as a novel food pursuant to regulation (EU) 2015/2283. EFSA J 2021;19:e06938. 10.2903/j.efsa.2021.6938 34987622 PMC8693246

[plaf068-B57] Usui T, Angert AL. Range expansion is both slower and more variable with rapid evolution across a spatial gradient in temperature. Ecol Lett 2024;27:e14406. 10.1111/ele.14406 38491734

[plaf068-B58] Vávra M, Špaček J, Koutecký P et al Asian *Wolffia globosa* (Roxb.) Hartog & Plas (Araceae) in bohemian wetlands—a new macrophyte for Czechia. Biologia 2024;79:1139–45. 10.1007/s11756-023-01413-7

[plaf068-B59] Visual Crossing Corporation . *Visual crossing weather 2024*, 2024. https://www.visualcrossing.com/ (25 February 2025, date last accessed).

[plaf068-B60] Vu GTH, Fourounjian P, Wang W et al Future prospects of duckweed research and applications. In: Cao XH, Fourounjian P, Wang W (eds.) The Duckweed Genomes. Cham: Springer International Publishing, 2020, 179–85.

[plaf068-B61] Waqas M, Naseem A, Humphries UW et al A comprehensive review of the impacts of climate change on agriculture in Thailand. Farm Syst 2025;3:100114. 10.1016/j.farsys.2024.100114

[plaf068-B62] Wooliver R, Vtipilthorpe EE, Wiegmann AM et al A viewpoint on ecological and evolutionary study of plant thermal performance curves in a warming world. AoB PLANTS 2022;14:plac016. 10.1093/aobpla/plac016 35615255 PMC9126585

[plaf068-B63] Ziegler P, Adelmann K, Zimmer S et al Relative in vitro growth rates of duckweeds (Lemnaceae)—the most rapidly growing higher plants. Plant Biol 2015;17:33–41. 10.1111/plb.12184 24803032

[plaf068-B64] Ziegler P, Appenroth KJ, Sree KS. Survival strategies of duckweeds, the world’s smallest angiosperms. Plants 2023;12:2215. 10.3390/plants12112215 37299193 PMC10255263

