# Supplementary material for: Thermal performance of Wolffia globosa under climate change: heatwaves impair population growth
Source: AoB Plants. 2026 Mar 11;18(2):plaf068. doi: 10.1093/aobpla/plaf068 (PMC12976596; doi:10.1093/aobpla/plaf068)
Supplement: plaf068_Supplementary_Data [file plaf068_supplementary_data.docx]

**Supporting Information 1:**

**Table S1:** Daily temperatures from a heatwave in Thailand. Data obtained from Visual Crossing Corporation (2024).

| **Date** | **Minimum (ºC)** | **Maximum (ºC)** | **Mean (ºC)** |
| --- | --- | --- | --- |
| 4/22/2024 | 30 | 37.5 | 33.3 |
| 4/23/2024 | 30.2 | 37.5 | 33 |
| 4/24/2024 | 30 | 37.7 | 32.9 |
| 4/25/2024 | 29.7 | 38.7 | 33.1 |
| 4/26/2024 | 29.2 | 37.5 | 32.9 |
| 4/27/2024 | 30 | 38.2 | 33.3 |
| 4/28/2024 | 30.4 | 37.7 | 33.2 |
| 4/29/2024 | 30.4 | 38.5 | 33.4 |
| 4/30/2024 | 30.6 | 40 | 33.8 |
| 5/1/2024 | 31 | 38.2 | 33.9 |
| 5/2/2024 | 31 | 38 | 33.5 |
| 5/3/2024 | 30 | 38.5 | 33.5 |
| 5/4/2024 | 30.1 | 36.7 | 32.7 |
| 5/5/2024 | 30 | 39.1 | 33.4 |
| 5/6/2024 | 29.8 | 37.4 | 32.8 |
| 5/7/2024 | 24.7 | 31.4 | 27.2 |
| 5/8/2024 | 25.1 | 35.9 | 30.1 |
| 5/9/2024 | 28.4 | 35.6 | 31.1 |
| 5/10/2024 | 26.9 | 35.7 | 30.8 |
| 5/11/2024 | 26.7 | 33.4 | 29.8 |
| 5/12/2024 | 26.9 | 36.6 | 30.3 |
| 5/13/2024 | 25 | 34.6 | 29.3 |
| 5/14/2024 | 25.3 | 35.8 | 30.2 |
| 5/15/2024 | 28.2 | 36.7 | 32.1 |
| 5/16/2024 | 28 | 34.1 | 30.1 |
| 5/17/2024 | 28.8 | 35 | 31.3 |
| 5/18/2024 | 28.2 | 33.7 | 30.9 |
| 5/19/2024 | 25.9 | 34.3 | 30.3 |
| 5/20/2024 | 26.2 | 35.7 | 30.4 |
| 5/21/2024 | 26.4 | 33.1 | 29.4 |
| 5/22/2024 | 26.6 | 34 | 29.6 |
| 5/23/2024 | 26.2 | 30.5 | 28 |
